# Internet Penetration and Leisure Activity Entropy: A Macro-Micro Integrated Analysis

**DOI:** 10.3390/e28020209

**Published:** 2026-02-11

**Authors:** Hanzun Li, Jianhua Dai

**Affiliations:** Business School, China University of Political Science and Law, Beijing 100088, China; 2502060235@cupl.edu.cn

**Keywords:** internet penetration, leisure activities, information entropy, nonlinear effects, macro-micro integrated analysis

## Abstract

Amid debates over internet penetration’s impact on leisure diversity—“macro-level entropy increase” vs. “micro-level entropy reduction”—this study explores their intrinsic link by introducing Shannon’s information entropy theory and constructing a three-tier framework (“micro-individual decision-making—macro-regional growth—macro–micro linkage”). Using microdata from the China General Social Survey and macro data from the China Economic and Financial Research Database, we adopt a multi-method approach (benchmark regression, mediation/nonlinear analysis) to test hypotheses. Key findings: micro-level internet penetration boosts individual leisure entropy; macro-level impact may follow an inverted U-shape, mediated by micro-level internet use; the entropy-increasing effect is strongest for learning-oriented leisure, weakest for social-oriented leisure; education, income, and internet penetration are core configurational conditions. This study contributes a quantitative leisure diversity framework, an integrated macro–micro model, and insights into the nonlinearities of internet penetration.

## 1. Introduction

Against the backdrop of the digital economy reshaping global production and living paradigms, internet penetration has extended from the industrial level to the core domains of individual daily behavior, becoming a key variable in reshaping social activity patterns and residents’ well-being [1]. By 2023, the global internet user base had reached 5.4 billion, covering approximately 67% of the total population [2]. Its penetration into the leisure sector has been particularly pronounced: the internet has not only reconfigured the temporal and spatial boundaries of leisure activities but also transformed the supply logic of leisure services and the decision-making patterns of individual leisure behaviors [3]. As a core indicator for measuring the quality of social development and the utility of residents’ lives, “leisure activities” are defined as voluntary activities undertaken for enjoyment [4]. As a vital gauge of societal development quality and individual well-being, the evolution of leisure activities reflects both macro trends in consumption structures and micro-level individual behavioral choices. Notably, in the digital era, leisure activities exhibit a contradictory duality of “diversification expansion” and “monotonic confinement,” a phenomenon that aligns naturally with the “disorder-to-order” evolutionary patterns revealed by entropy theory [5]. Against this backdrop, clarifying the intrinsic connection between internet penetration and the entropic transformation of leisure activities is not only a core proposition for interpreting social behavioral evolution in the digital age but also a vital exploration for refining economic theory and optimizing public welfare.

Existing research on the internet and leisure activities has yielded two divergent conclusions anchored, respectively, to the independent dimensions of macro-industry and micro-behavior, yet lacking cross-dimensional integration. At the macro-industrial level, some studies indicate that internet-driven innovation in the software industry (e.g., leisure apps, online entertainment platforms) has significantly reduced the supply costs and access barriers of leisure services [6]. This has propelled leisure activities from an “offline-centric” model toward an “online–offline integrated” paradigm [7], fostering a leisure ecosystem spanning multiple domains such as knowledge-based subscriptions and virtual social interactions [8,9]. Consequently, the diversity of leisure activities and the richness of their formats have been markedly enhanced. From this perspective, the internet is viewed as a driver of “entropy increase” in leisure activities. By breaking the temporal and spatial monopolies of traditional leisure resources [10], it enhances the disorder and complexity of the leisure system, thereby expanding the boundaries of societal leisure welfare.

At the micro-individual level, however, another body of research presents contrasting conclusions: the internet’s attention-capturing mechanisms (such as algorithmic recommendations and instant messaging alerts) can induce path dependence in individual leisure behaviors. This traps certain groups in fragmented entertainment, manifested as leisure time dominated by single formats such as short videos and online games, while participation in traditional activities such as reading, outdoor sports, and face-to-face socializing significantly declines [11,12,13]. From this perspective, the internet is regarded as a key factor in the “reduction in entropy” within leisure activities: it tends to organize the leisure system by compressing individual choice space, but this organization often comes at the expense of leisure quality.

Although existing research has captured the dual impact of the internet on leisure activities, its core gaps and limitations remain prominent, highlighting the necessity of this study. First, the fragmentation of perspectives is pronounced. Macro studies predominantly focus on the expansion of leisure resources from the industry supply side, while micro studies are confined to the singular logic of individual behavioral choices. This creates an analytical barrier between “supply” and “demand,” failing to explain how macro-level internet industry penetration translates into micro-level individual leisure decisions. It also struggles to resolve the theoretical contradiction between “macro-level entropy increase” and “micro-level entropy reduction,” resulting in research conclusions that lack systematic coherence and consistency. Second, theoretical tools exhibit shortcomings. Existing research predominantly relies on traditional economic theories, failing to incorporate entropy theory for quantitative analysis of the “diversity-centralization” evolution in leisure activities. This prevents precise measurement of transitions between disorder and order within leisure systems and hinders the revelation of underlying dynamic mechanisms, confining studies to descriptive levels without essential interpretation of underlying patterns.

From a theoretical perspective, this study employs entropy theory as a core link to construct an integrated macro–micro dual-perspective analytical framework. This approach breaks through existing research barriers, provides new theoretical tools for interpreting the complex evolution of leisure activities, and expands the application scenarios of entropy theory in socioeconomic fields. In terms of practical value, this study explores the relationship between internet penetration and the evolution of leisure activities, providing theoretical foundations for government guidance on promoting healthy leisure practices and enhancing national utility levels.

Based on the aforementioned research context and the literature gaps, the core research questions of this study focus on three levels:

(1) At the macro-level, how does internet penetration influence the entropy change process of leisure activities? Does it exhibit a nonlinear “double-edged sword” effect? (2) At the micro level, what quantitative relationship exists between individual internet usage characteristics and the entropy evolution of leisure activities? Does the entropy-increasing effect vary across different types of leisure activities? (3) Is there a transmission pathway between macro-level internet penetration and micro-level entropy evolution in leisure behavior? How does this linkage mechanism operate?

To answer these questions, we employ multi-method empirical strategies (benchmark regression, mediation analysis, nonlinear regression, fuzzy-set qualitative comparative analysis) using microdata from the China General Social Survey (2010–2023) and macro data from the China Economic and Financial Research Database. Key findings: 1. At the micro level, internet usage significantly increases individual leisure entropy, with education and income amplifying this effect. 2. At the macro-level, internet industry development exerts an inverted U-shaped influence on regional leisure entropy. 3. Macro-level internet penetration transmits leisure entropy through micro-level internet usage. 4. Internet penetration has the greatest entropy-increasing effect on learning-related leisure activities, followed by relaxation-related leisure, while its effect on social-related leisure is the smallest. 5. Education, income, and internet penetration constitute the core configurational conditions for increasing leisure entropy.

The subsequent structure of this paper is as follows: Section 2 constructs a theoretical model, establishing a dual-perspective analytical framework based on entropy theory, macroeconomic growth theory, and microbehavioral decision theory, and proposes testable core hypotheses. Section 3 outlines the research design, specifying the overall research framework, data collection and processing methods, and models employed to provide methodological support for hypothesis testing. Section 4 analyzes empirical results, presenting regression outcomes and robustness tests for both macro and micro data to validate foundational assumptions. It reveals core macro–micro linkage pathways and interprets the differentiated impact of internet penetration on various leisure activities. Section 5 concludes with a discussion and findings, summarizing key research insights, articulating theoretical contributions and practical implications, while acknowledging study limitations.

## 2. Theoretical Model Construction

This section centers on Shannon’s information entropy theory as the core link, integrating micro-level individual behavioral decision-making theory with macroeconomic growth theory to construct a three-tiered analytical framework: “micro-individual—macro-regional—macro–micro linkage.” It first defines the measurement logic of leisure activity entropy, then separately derives the operational mechanisms of internet penetration at micro- and macro-levels. Finally, it constructs macro–micro transmission pathways and proposes testable research hypotheses based on theoretical deduction.

### 2.1. Shannon’s Information Entropy Theory and the Information Entropy of Leisure Activities

Shannon information entropy was first introduced by Claude E. Shannon in 1948 in his paper “A Mathematical Theory of Communication” [14]. Its core principle lies in measuring the information content and degree of disorder within a system through the uncertainty of probability distributions. Its fundamental expression is as follows:

(1) $$H\left(x\right)=-\sum_{x\in X}P\left(x\right){log}_{2}\left[P\left(x\right)\right]\;$$

Here, x represents a discrete random variable, and P(x) denotes the probability of the variable assuming a particular state. A higher value of H(x) indicates greater uncertainty and diversity in the system’s state. Originally applied in the field of communications, this theory has since been extensively extended to socioeconomic systems. It is used to analyze the diversity and complexity of resource allocation and behavioral choices [15,16], providing a suitable theoretical tool for quantifying the “diversity-centralization” characteristics of leisure activities.

In leisure economy research, individuals’ time allocation across different leisure activities fluctuates significantly within a given time unit. This highly volatile behavior can be described using the probability distribution P(x) [17]. In the digital era, leisure activities exhibit both “diversification expansion” and “monotonic locking.” Factors such as AI technological innovation, social stratification, and cultural diversity have broadened the range of leisure choices, leading to a “diversification expansion” characteristic in leisure activities [18]. Simultaneously, factors such as algorithmic echo chambers and behavioral path dependence drive convergence in leisure behaviors, manifesting as “monotonic locking.” The “diversity-centralization” pattern of leisure activities aligns closely with Shannon entropy’s “disorder-to-order” evolutionary principle. Previous studies have employed either the number of leisure activities participated in or the proportion of time spent on a single activity to measure leisure diversity. However, simply using the number of leisure activities only reflects the breadth of leisure types and fails to capture the balance of time allocation across different activities. Similarly, the proportion of time spent on a single activity can only gauge the concentration of leisure behavior but cannot compare the overall diversity level when multiple activities coexist. Shannon entropy, calculated based on the full-dimensional probability distribution, simultaneously captures both the number of activity types and the balance of time allocation. This effectively addresses the shortcomings of previous methods. Based on this, this paper constructs a measure function for the leisure entropy to quantify the diversity of leisure behavior.

Scholars often employ the temporal structure of leisure activities to measure their structural characteristics. Aguiar and Lee assess leisure quality through the allocation of leisure time [19,20]. Tsaur argues that the temporal structure and type of leisure activities are key prerequisites for leisure participation [21]. Building upon prior research, we construct a measure of individual leisure information entropy based on the type and temporal structure of leisure activities, as follows:

(2) $$S_{i}=-\sum_{k=1}^{K}p_{ik}{log}_{2}\left(p_{ik}\right)$$

In the equation, $p_{ik}=\frac{T_{ik}}{\sum_{k=1}^{K}T_{ik}}$ denotes the proportion of time individual i spends on leisure activity type k (where K is the total number of leisure activity types), and $T_{ik}$ represents the duration individual i participates in leisure activity type k. When $p_{ik}$ is distributed more evenly (i.e., individuals spend similar amounts of time on multiple leisure activities), the $H_{i}$ value increases, indicating greater diversity in leisure activities. When $p_{ik}$ = 1 (participating in only one type of leisure activity), $H_{i}$ = 0, signifying complete concentration of leisure activities.

Howard categorizes leisure activities into four types: outdoor-nature-based, sports-based, esthetic-cultured, and leisure-grooming [22]. The China General Social Survey (CGSS) is one of the most important scales for studying leisure activities. It subdivides leisure activities into fifteen categories: reading books/newspapers/magazines, physical exercise, watching TV/videos, watching movies, shopping, attending concerts/exhibitions, listening to music, watching sports events, performing crafts, family gatherings, social gatherings with friends, and internet-based leisure. This framework effectively measures the types and frequency of residents’ leisure activities. Zhang conducted confirmatory factor analysis on the CGSS, identifying four leisure activity categories: hobby/social, fitness, learning, and entertainment [23]. This classification aligns with Howard’s four-category framework. Subsequently, the CGSS’s leisure measurement has been widely adopted by scholars, such as Yue’s study on elderly leisure patterns and Jia’s research on elderly leisure participation [24,25]. We defined leisure activity categories based on CGSS scales to derive leisure entropy. Since internet leisure is highly correlated with internet penetration, we excluded internet leisure to eliminate confounding effects. We then assessed diversity levels across the remaining fourteen leisure activities to specify leisure entropy metrics. With the number of leisure activities k fixed, the weights and values of leisure entropy are solely determined by individuals’ reported time spent on leisure activities T_ik_.

To extend individual-level entropy values to the regional level, this paper further constructs regional leisure entropy to quantify the overall diversity of leisure activities within a region. Its benchmark equation is as follows:

(3) $$S_{r}=-\frac{1}{N_{r}}\sum_{i=1}^{N_{r}}\sum_{k=1}^{K}p_{ik,r}\log_{2}p_{ik,r}$$

Given the complexity of the operation, Equation (A3) can be simplified to the mean form of the microscopic individual’s leisure entropy:

(4) $$S_{r}=\frac{1}{N_{r}}\sum_{i=1}^{N_{r}}S_{i}$$

Here $N_{r}$ denotes the number of sample individuals in region r, and $p_{ik,r}$ represents the proportion of time individual i spends on the kth type of leisure activity within the region. The core concept of regional leisure entropy is that higher entropy values indicate greater average diversity in residents’ leisure behaviors within the region, reflecting stronger inclusiveness and balance in regional leisure resources.

The introduction of Shannon entropy into leisure activity analysis requires three fundamental assumptions, whose validity is supported by both theoretical and practical logic: 1. Probability Distribution Assumption. The allocation of leisure time by individuals/regions satisfies probability normalization ($\sum_{k=1}^{K}P_{ik}=1$, where $P_{ik}$ represents the proportion of time individual i spends on the kth type of leisure activity, and K denotes the total number of leisure activity types), conforming to Shannon entropy’s basic constraints on probability distributions. In practice, the total daily leisure time per individual is fixed, and the sum of time shares across all leisure categories must equal 1, providing an objective empirical basis for this assumption. 2. Diversity preference assumption. Individuals possess an endogenous preference for leisure entropy, meaning increased leisure entropy directly enhances individual utility. This aligns with behavioral economics’ “diversity seeking theory.” 3. System Openness Assumption. The leisure system is an open system capable of achieving entropy change through material and information exchange with the external environment [26]. In the digital age, external factors such as internet technology and new leisure services continuously flow into the leisure system, breaking the limitations of Shannon entropy in closed systems and aligning with the evolutionary characteristics of leisure behavior.

Building upon this foundation, this paper further elucidates the entropy change logic of leisure systems.

The core of Shannon’s information entropy lies in “entropy change” (entropy increase or decrease). The primary driver of leisure entropy increase is the input of external leisure resources, with the specific pathway being that increased input of external leisure resources expands the variety of leisure activities, leading to more evenly distributed time allocation probabilities and thereby enhancing the information entropy of leisure activities. For instance, the introduction of virtual reality technology creates novel leisure services (e.g., virtual reality tourism) [27], broadening the leisure choice set and dispersing time allocation, ultimately achieving an increase in leisure entropy.

In Shannon’s information entropy, signal homogenization within a system reduces uncertainty. The core driver of leisure entropy reduction is individual/regional path dependence in leisure activities. The specific pathway is as follows: Leisure path dependence increases the probability of individuals engaging in a single leisure activity while decreasing the probability of other leisure activities, ultimately reducing the information entropy of leisure activities. For example, an individual’s addiction to online gaming leads to highly concentrated leisure time, compressing the time allocated to social interaction activities, shrinking the leisure choice set, making time allocation more concentrated, and ultimately resulting in a decrease in leisure entropy.

When the entropy-increasing effect of external resource inputs balances the entropy-reducing effect of internal path dependencies, leisure entropy reaches a steady state ($\frac{dH}{dt}=0$, where t represents time). The steady-state value of leisure entropy represents the “optimal diversity level” of the leisure system—avoiding both “leisure fragmentation caused by excessive diversity” and “leisure homogenization resulting from excessive concentration.”

### 2.2. Core Mechanisms and Research Hypotheses

#### 2.2.1. Micro Level Internet Penetration and Leisure Entropy

At the micro level, scholars primarily focus on the internet’s impact on vulnerable groups, yet few have systematically explored its systemic influence on diversifying leisure activities. Simsek and Çevik examine leisure among the elderly, arguing that the internet reduces the cost of engaging in leisure activities for this demographic [28]. Roderick notes that niche groups and individuals with lower social status utilize the internet more frequently for leisure [29]. Kadir identifies emotional loneliness as a significant cause of online gaming disorders [30].

Building upon existing research, we construct a micro-level behavioral decision model based on behavioral economics’ “diversity-seeking theory” (as shown in Appendix A), revealing the following impact mechanisms [31,32]: 1. Increased internet usage intensity significantly elevates an individual’s leisure entropy. The internet enhances leisure diversity through cost-reduction and accessibility-expansion effects, while on one hand, it lowers barriers to participation in niche leisure activities; on the other, one-stop leisure platforms broaden the range of leisure options. 2. Higher education and income levels amplify the Internet’s entropy-increasing effect on leisure activities. Increased education strengthens individual diversity preferences, while higher income eases budget constraints on high-cost leisure, both reinforcing the Internet’s role in promoting leisure entropy. 3. The internet’s entropy-increasing effect is significantly stronger for relaxation-oriented leisure than for social leisure. Relaxation-oriented leisure adapts better to online formats, as the internet substantially lowers participation costs and time barriers. In contrast, the core value of social leisure stems from offline interaction. The internet’s substitution effect may reduce the time allocated to offline socializing, limiting its potential for diversity enhancement. Thus, we propose the following hypotheses:

**H1.**  *At the micro level, internet penetration significantly and positively influences the entropy value of individual leisure activities, meaning that higher internet penetration correlates with greater diversity in individual leisure activities.*

**H2.**  *At the micro level, the entropy-increasing effect of internet penetration on relaxation-oriented leisure activities is significantly higher than that on social-oriented leisure activities.*

#### 2.2.2. Macro-Level Internet Penetration and Leisure Entropy

At the macro-level, scholars have focused on the direct expansion of leisure activities facilitated by the internet, yet they have rarely explored the systemic impact of internet penetration on leisure activities. Chen examined leisure satisfaction derived from online gaming as a leisure activity [33]. Mohammad investigated the influence of internet-based leisure on employee satisfaction [34].

Building upon existing research, we refine neoclassical growth theory using feasible capability theory [35]. By incorporating leisure entropy into the social welfare function [36], we construct a macroeconomic growth model (as shown in Appendix A), revealing the following impact mechanisms:

1. Regional internet industry development drives leisure entropy growth through two pathways. One via technological spillover effects, where regional internet industry development increases residents’ income and relaxes budget constraints on leisure choices; the other via supply expansion effects, enriching regional leisure options through innovative digital leisure services. 2. In the later stages of internet industry development, platform monopolies may lead to service homogenization, reversing the entropy increase effect and forming an inverted U-shaped relationship. Thus, we derive the following hypotheses:

**H3.**  *At the macro-level, regional internet industry development significantly and positively influences regional leisure entropy, meaning higher levels of internet industry development correlate with greater overall diversity (entropy) in regional leisure activities.*

**H4.**  *At the macro-level, the impact of internet industry development on regional leisure entropy follows an inverted U-shaped relationship. Specifically, during the early stage of development (when embedded software revenue is below a threshold), it increases regional leisure entropy, while in the later stage (when embedded software revenue exceeds the threshold), it decreases regional leisure entropy.*

#### 2.2.3. Macro–Micro Linking Mechanism

At the macro–micro linkage mechanism level, scholars have reached preliminary conclusions that macro-level industrial development drives down internet usage costs and increases individual internet usage frequency [37,38]. However, they have yet to connect macro-level industrial development with micro-level leisure activities. Neoclassical supply–demand transmission theory and industrial organization theory suggest that macro-level internet penetration may influence leisure entropy through a one-way transmission system: “macro-supply → micro-constraints → decision feedback → macro-steady state” (as illustrated in Appendix A). This involves both direct and indirect effects: 1. Direct effect: Technological spillovers from internet industry development directly enrich regional leisure diversity; 2. Indirect effect: Macro-level industrial development reduces internet usage costs, increases individual internet usage frequency, and consequently impacts individual leisure entropy, aggregating to the regional level. Thus, we formulate the following hypothesis:

**H5.**  *Macro-level internet industry development indirectly influences leisure activity entropy through the mediating effect of micro-level internet penetration, meaning micro-level internet penetration serves as the mediating variable for macro-level internet penetration’s impact on leisure entropy.*

#### 2.2.4. Theoretical Framework Integration

**Figure 1:**
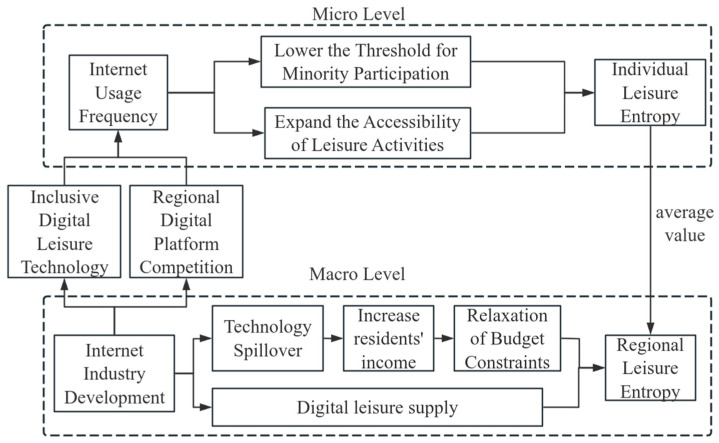
Schematic Diagram of the Pathways Through Which the Internet Influences Leisure Entropy (Arrows denote influence paths).

In summary, the theoretical framework constructed in this paper can be integrated into a “three-tiered pathway,” as illustrated in Figure 1: At the micro level, internet usage enhances individual leisure entropy by reducing costs and expanding accessibility. At the macro-level, internet industry development drives regional leisure entropy growth through technological spillovers and supply expansion. At the macro–micro linkage level, macro-level internet penetration facilitates entropy transmission by relaxing micro-level usage constraints, exhibiting a double-edged sword effect with an inverted U-shaped relationship at the macro-level.

## 3. Materials and Methods

To validate the five research hypotheses proposed earlier, this section constructs a hierarchical research design framework encompassing “micro-individual—macro-regional—macro–micro linkage.” It clarifies data sources and processing workflows, model configuration logic, algorithm implementation details, and evaluation metrics.

### 3.1. Overall Research Design

This study simultaneously addresses three core questions: “The impact of micro-level internet usage on individual leisure entropy,” “The role of macro-level internet industries in regional leisure entropy,” and “The macro-micro transmission mechanism.” Therefore, a multi-method research design is adopted, with the suitability of each method for the research questions outlined below:

In this study, empirical tests are performed using SPSS 25. We employ benchmark regression models to test linear relationships at the micro (H1, H2) and macro (H3) levels. OLS regression quantifies the marginal effects of core variables and conducts robustness tests. A mediation model verifies the transmission pathway linking macro and micro levels (H5), using three-stage regression to decompose direct and indirect effects for “mechanism identification.” This study incorporates quadratic terms for core explanatory variables, employing a nonlinear regression model to test the inverted U-shaped effect of macro-level internet penetration on regional leisure entropy (H4), addressing the need to validate “nonlinear relationships.”

### 3.2. Data Collection and Processing

#### 3.2.1. Data Source

- Micro-level individual data

This study integrates data from nine waves of the China General Social Survey (CGSS) conducted between 2010 and 2023 (2010, 2011, 2012, 2013, 2015, 2017, 2018, 2021, 2023), maintained by the China Survey and Data Center at Renmin University of China. The data encompass socioeconomic characteristics, leisure behaviors, and internet usage among residents across 31 provincial-level administrative regions in mainland China. The initial sample size was 96,417. After removing cases with missing core variables, a valid sample of 62,838 cases was obtained. Relevant data supporting this study are openly available in Mendeley Data at DOI:10.17632/c75kyhzbp9.2, accessed on 17 December 2025.

- Macro Regional Data

This study aggregates nine waves of microdata into provincial-level panel data (2010–2023, comprising 234 provincial-year observations) based on the provincial administrative regions to which the micro samples belong. For missing micro-level data, the China Social Science Research Database (CSMAR) serves as the supplementary data source. Macro-level indicators such as embedded software revenue, per capita disposable income, regional GDP, and year-end population figures are drawn from the National Bureau of Statistics of China and the China Economic and Financial Research Database (CSMAR). Relevant data supporting this study are openly available in Mendeley Data at DOI:10.17632/c75kyhzbp9.2, accessed on 17 December 2025.

#### 3.2.2. Variable Measurement and Operationalization

- Core Explanatory Variable: Internet Penetration

At the micro level, we employ the response to the CGSS questionnaire item “Frequency of Internet Use in the Past Year” as the representative variable for Internet penetration. The response options are “Never (1), Rarely (2), Sometimes (3), Often (4), Very Frequently (5),” forming a continuous ordinal variable. This variable is subsequently Z-score standardized in subsequent analyses and denoted as $I_{i}^{\rho -1}$.

At the macro-level, we use the provincial-level “embedded software revenue” indicator as the representative variable for internet penetration. Data is sourced from the CSMAR database, undergoes Z-score standardization, and is denoted as $I_{i}$.

- Core explanatory variable: Leisure activity entropy

The measurement of leisure activity entropy is based on Shannon’s information entropy theory, comprising two distinct systems: the fundamental entropy value and the robustness test entropy value. The specific steps are as follows:

At the micro level, we use the CGSS questionnaire result “In the past year, did you frequently engage in the following activities during your free time?” to calculate the entropy of leisure activities. The items encompass 12 activity categories, which we grouped into three types: Learning (reading books/newspapers/magazines, physical exercise), Relaxation (watching TV/videos, watching movies, shopping, attending concerts/exhibitions, listening to music, watching sports events, performing crafts), and Socializing (family gatherings, friend gatherings).

We converted activity frequency options into daily occurrence probabilities, assigning values based on the median time proportion: “Never” was assigned a value of 0, ‘Daily’ was assigned 1; “Several times a week” was assigned 0.571 (mean of $[\frac{1}{7}-1)$; “Several times a month” was assigned 0.088 (mean of $[\frac{1}{30}-\frac{1}{7})$); and “Several times a year or less” was assigned 0.018 (mean of $\left(\frac{1}{365}-\frac{1}{30}\right)$). This yields the probability of engaging in a particular leisure activity during a day. Assuming individuals engage in only one leisure activity at a time, we calculate the probability of an individual participating in a specific leisure activity at any given moment by weighting the probability of that activity occurring relative to the total probability of all leisure activities occurring. This probability is then substituted into Equation (A2) to compute leisure activity entropy. Leisure activity entropy uses Z-score standardized data, denoted as $S_{i}^{\rho -1}$.

At the macro-level, based on Equation (A4), we calculate the regional leisure entropy value using the mean of individual leisure entropies within the region. This value is Z-score standardized and denoted as $ES_{r}$.

Since leisure activity entropy has been rarely studied, we employed two datasets to calculate the entropy values of leisure activities, using the entropy data from the second dataset for robustness testing. We calculated the second set of leisure activity entropy using the CGSS item “Leisure Activity Types” (comprising three categories: learning-oriented leisure, relaxation-oriented leisure, and social-oriented leisure). We applied the same probability weighting and entropy calculation methods as above to ensure measurement reliability. The second set of leisure activity entropy was Z-score standardized and denoted as ${S2}_{i}^{\rho -1}$.

- Control variable

At the micro level, we control for birth year (Year), education level (Education, scored 1–8), annual total income (Income), political participation (Politics, scored 1–4), life satisfaction (Happiness, scored 1–5), and social relative status (Social status, scored 1–10) and physical health (Health, scored 1–5), and participation in religious activities (Heligion, scored 1–9). At the macro-level, we additionally controlled for per capita disposable income (pre-Income), regional GDP (GDP), and year-end population (People). All control variables were Z-score standardized to eliminate differences in measurement units.

Based on the derivation of micro-level individual behavioral decision models, educational attainment and income levels influence the entropy-increasing effect of leisure activities, prompting their inclusion as controls in this study. Similarly, according to macroeconomic growth model derivations, per capita income (a proxy variable for per capita capital stock $k_{r}$), regional GDP (Proxy variable for regional economic output $Y_{r}$), and population size (Proxy variable for labor input $L_{r}$) affect the entropy-increasing effect of leisure activities, justifying their inclusion as controls. In microeconomics, the utility function was originally conceived to represent individual well-being. However, due to the difficulty in quantifying unit well-being, scholars later adopted consumption opportunities as a measure of well-being, with income gradually replacing well-being as the metric for utility [39]. As the utility function forms the foundation of micro-level individual decision-making models, this paper traces the essence of utility, controls for happiness and its influencing factors, and explores the relationship between the internet and leisure entropy within the micro-level individual decision-making framework. Age [40], level of political participation [41], social relative status [42], health status [43], and participation in religious activities are key determinants of happiness [44]. Therefore, this study also controls for these variables. Among these, life satisfaction and religious participation may be influenced by leisure activities, potentially confounding regression results. To address potential over-control issues and ensure robustness, we conducted the baseline regression in three stages: first, without any control variables, then including all control variables except life satisfaction and religious participation, and finally incorporating all control variables.

- Replacement of variables

To eliminate bidirectional causality between the independent and dependent variables, we employ prior internet penetration (I_i1_) and fiber-optic cable length (I_i2_) as instrumental variables for internet penetration in our endogeneity tests. Past internet penetration is measured using the internet penetration from two years prior. For example, 2015’s internet penetration is used to measure 2017’s past internet penetration, serving as an instrumental variable for 2017’s internet penetration. Fiber-optic cable length data is sourced from the National Bureau of Statistics of China. As internet infrastructure, fiber-optic cable length is minimally influenced by specific internet usage activities, making it suitable as an instrumental variable for internet penetration. Due to the absence of fiber-optic cable length data for 2010 in the National Bureau of Statistics of China, we conducted the endogeneity test using data from 2011 to 2023 only.

### 3.3. Model and Algorithm Description

#### 3.3.1. Baseline Regression Model

- Macro Benchmark Model

Based on the dual pathway of enhancing regional leisure entropy through the development of the internet industry, the following equation can be derived:

(5) $$ES_{r}=f(I_{i},Z)$$

Among these, $ES_{r}$ represents the entropy value of leisure activities, $I_{i}$ denotes the development of the internet industry, and Z signifies other influencing factors.

To validate the impact of internet industry development on the entropy value of leisure activities, this study establishes the following benchmark model for macro-level research:

(6) $$ES_{r}=\alpha_{1}+\beta_{1}I_{i}+\mu \sum Z+\varepsilon_{1}$$

Among these, α_1_ represents the constant term, ε denotes the random disturbance term, and the value of β_1_ is used to examine the magnitude of the impact of internet industry development on the entropy value of leisure activities. A significant positive value would support H3. This study employs SPSS 25 software to validate the macro-level benchmark model, micro-level benchmark model, nonlinear model, and mediation effect model.

- Micro benchmark model

The benchmark model for micro-level research is as follows:

(7) $$S_{i}^{\rho -1}=\alpha_{4}+\beta_{4}I_{i}^{\rho -1}+\mu \sum Z+\varepsilon_{4},\beta_{4}=\frac{\partial T_{ik}*\left(1-\omega_{i}\right)}{\omega_{i}\partial S_{i}}$$

Among these, α_4_ represents the constant term, ε_4_ denotes the random disturbance term, and the value of β_4_ is used to examine the magnitude of the impact of internet usage frequency on the entropy value of leisure activities. A significant positive value supports H1.

To verify whether the entropy-increasing effect of the internet on relaxation-oriented leisure activities (e.g., watching movies, listening to music) is significantly higher than that on social-oriented leisure, this study establishes separate models to measure the entropy-increasing effects of the internet on relaxation-oriented leisure and social-oriented leisure. The resulting micro-level model is as follows:

(8) $${S_{i}^{social}}^{\rho -1}=\alpha_{5}+\beta_{5}{I_{i}^{social}}^{\rho -1}+\mu \sum Z+\varepsilon_{5}$$

(9) $${S_{i}^{relax}}^{\rho -1}=\alpha_{6}+\beta_{6}{I_{i}^{relax}}^{\rho -1}+\mu \sum Z+\varepsilon_{6}$$

By comparing the magnitudes of β_5_ and β_6_ values, we examine the extent to which internet usage frequency influences the entropy value of leisure activities. If β_6_ > β_5_, this supports H2.

#### 3.3.2. Nonlinear Model

To verify whether the development of the internet industry has a double-edged sword effect on leisure activities, this study establishes the following nonlinear model of internet industry development:

(10) $$ES_{r}=\alpha_{3}+\beta_{2}I_{i}+\beta_{3}I_{i}^{2}+\mu \sum Z+\varepsilon_{3}$$

Among these, α_3_ represents the constant term, and ε_3_ denotes the random disturbance term. The direction of β_2_ and β_3_ values is used to determine whether the development of the internet industry exerts a double-edged sword effect on leisure activities. If β_2_ is significantly positive and β_3_ is significantly negative, this supports H4.

#### 3.3.3. Mediation Effect Model

This study employs a three-stage mediation model to examine the unidirectional transmission system of “macro-level supply → micro-level constraints → decision feedback → macro-level steady state,” verifying Hypothesis H5 as follows:

The first stage investigates the impact of macro-level internet penetration on micro-level internet usage, as shown in Equation (A11).

(11) $${I_{i}^{social}}^{\rho -1}=\alpha_{7}+\beta_{7}I_{i}+\mu \sum Z+\varepsilon_{7}$$

The second stage examines the direct impact of macro-level internet penetration on regional leisure entropy (i.e., the macro-level baseline model), as shown in Equation (A6).

The third stage investigates the total effect model incorporating the mediating variable (micro-level internet usage), as depicted in Equation (A12).

(12) $$ES_{r}=\alpha_{8}+\beta_{8}I_{i}+\beta_{8}{I_{i}^{social}}^{\rho -1}+\mu \sum Z+\varepsilon_{8}$$

By comparing the differences between β1 and β8 values, the coefficient of the mediating effect can be determined.

#### 3.3.4. Endogeneity Test

This paper examines the causal relationships among variables by conducting endogeneity tests to explore the possibility of mutual causality between them. The diversity of leisure activities may foster more internet-dependent leisure pursuits. We set the length of optical fiber lines and past internet penetration as instrumental variables for internet penetration to conduct this test.

Since the CGSS typically conducts surveys every two years, we set the lag period to two years. We use internet penetration two years prior as the explanatory variable and leisure activity entropy two years later as the dependent variable. This approach eliminates the possibility that leisure activity entropy influences internet penetration. If the results remain significant after replacing the dependent variable with internet penetration, we tentatively conclude that the causal relationship between the variables is that internet penetration affects leisure activity entropy.

The entropy value of internet-related leisure activities may influence residents’ internet usage patterns. However, regardless of how residents utilize the internet, it is difficult for their usage to directly impact the underlying internet infrastructure. As internet infrastructure, the length of fiber optic cable remains unaffected by residents’ leisure activities yet constitutes a prerequisite for internet usage. Fiber optic cable length is highly correlated with the independent variable but unrelated to the dependent variable. Therefore, we employ fiber optic cable length as the explanatory variable to test its impact on leisure activity entropy. If the results remain significant after replacing the explained variable with fiber optic cable length, we conclude that the causal relationship between variables is that internet penetration influences leisure activity entropy.

## 4. Results

### 4.1. Stylized Facts Analysis

#### 4.1.1. Descriptive Statistics of Variables

Table 1 presents the sample characteristics of our variables. The mean for macro-level regional leisure entropy is 0.569 (standard deviation 0.076), while the micro-level leisure entropy mean is 0.570 (standard deviation 0.210). Macro-level embedded software revenue averaged 198.499 (standard deviation 494.797), and micro-level internet usage frequency averaged 2.560 (standard deviation 1.672). Given the significant differences in the dimensions of these variables, all were Z-score standardized to eliminate the interference of dimensionality on the regression results.

#### 4.1.2. Preliminary Analysis of the Relationship Between Internet Penetration Rate and Regional Leisure Entropy

**Figure 2:**
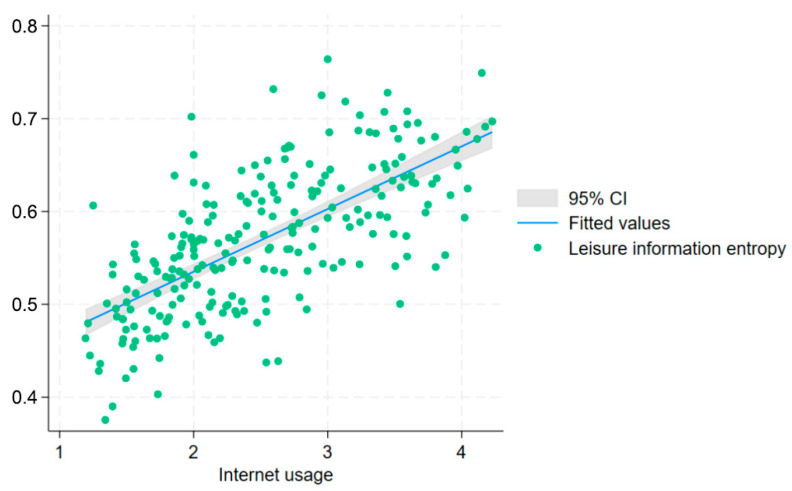
Relationship between Internet penetration and leisure activity entropy at the macro-level.

As shown in Figure 2, within the sample period, regional leisure entropy and the frequency of internet use exhibit a trend of moving in the same direction. As internet usage increases, regional leisure entropy shows an overall upward trend, indicating a preliminary positive correlation between the two.

### 4.2. Micro-Level

As shown in Table 2, at the micro level, regardless of whether other variables are controlled, the frequency of internet use significantly increases the entropy value of individuals’ leisure activities, supporting the validity of H1. Without controlling for variables, the regression coefficient for the frequency of internet use was 0.552 (*p* < 0.001). After incorporating control variables, the coefficient decreased to 0.372 (*p* < 0.001), with a model fit index of 0.384, explaining approximately 38% of the variation in individual leisure activity entropy. Furthermore, income (coefficient: 0.008, *p* = 0.018) and educational attainment (coefficient: 0.248, *p* < 0.001) exerted significant positive effects on leisure activity entropy.

### 4.3. Macro-Level

Table 3 presents the results of the macro-level benchmark regression. At the macro-level, embedded software revenue significantly increases regional leisure entropy regardless of whether other variables are controlled, supporting the validity of H3. Without control variables, the regression coefficient for embedded software income is 0.247 (*p* < 0.001). After incorporating control variables, the coefficient decreases to 0.107 (*p* = 0.043), with a model fit index of 0.770 explaining 77% of regional leisure entropy variation. At the macro-level, the regression coefficients for per capita disposable income (coefficient: −0.102, *p* = 0.034) and life satisfaction (coefficient: −0.137, *p* = 0.000) were significantly negative. However, the coefficients for regional GDP (coefficient: 0.036, *p* = 0.694) and end-of-year population (coefficient: −0.072, *p* = 0.282) failed to pass the significance test.

### 4.4. Robustness Test and Endogenous Test

At the micro level, we conducted robustness tests using the second set of leisure entropy data (see Table 4). The coefficient for internet usage intensity was 0.289 (*p* < 0.001) without controlling variables and 0.115 (*p* < 0.001) with controlling variables. The direction and significance of the results remained unchanged, validating the robustness of the benchmark regression findings.

At the macro-level, we employ the lagged internet penetration rate and fiber-optic cable length as instrumental variables for internet penetration in our analysis (see Table 4). As shown in Table 5, after treating internet penetration as an instrumental variable, the direction and significance of the results remain unchanged. The benchmark regression results exhibit no endogeneity issues such as bidirectional causality.

### 4.5. Macro–Micro Linkage Mechanism Pathway

Table 6 reports the results of the three-stage mediation effect tests. In the first-stage regression, the coefficient for macro-level embedded software revenue on the micro-level frequency of internet use was −0.156 (*p* < 0.001). In the second-stage regression, the direct effect coefficient of macro-level embedded software revenue on regional leisure entropy was 0.107 (*p* = 0.211). In the third-stage regression, after including the micro-level frequency of internet use, the coefficient for macro-level embedded software revenue was 0.140 (*p* = 0.010), and the coefficient for the micro-level frequency of internet use was 0.210 (*p* = 0.019).

As shown in Table 7, the mediation effect decomposition revealed a direct effect of 0.140 (95% CI: 0.033 to 0.246) and an indirect effect of −0.033 (95% CI: −0.076 to −0.005). with a total effect of 0.107 (95% CI: 0.003 to 0.211). The negative mediating effect accounted for 30.8% of the total effect. Hypothesis H5 was supported.

### 4.6. Further Analysis

#### 4.6.1. The Inverted U-Shaped Impact of Internet Penetration at the Macro-Level

Table 8 reports the nonlinear regression results, indicating a U-shaped effect of the internet at the macro-level on the entropy value of leisure activities, confirming hypothesis H4. After incorporating control variables, the coefficient for the linear term of embedded software revenue is 0.400 (*p* < 0.001), while the quadratic term coefficient is −0.066 (*p* = 0.004). The model’s goodness-of-fit R^2^ improves to 0.779. Calculations reveal the inverted U-shaped inflection point at $I_{i}^{*}=-\frac{0.400}{2\times (-0.66)}\approx 3.03$. Beyond this threshold, embedded software revenue’s positive impact on regional leisure entropy reverses to a negative effect.

#### 4.6.2. The Impact of Internet Penetration on the Entropy Values of Different Types of Leisure Activities

We examined how internet penetration affects the entropy values of different types of leisure activities, with the results shown in Table 9.

The heterogeneity test results are shown in Table 9. Internet penetration significantly affects all three types of leisure activities. The coefficient of influence on the entropy value of learning-oriented leisure activities is the largest, that on recreational leisure activities is moderate, and that on relaxation-oriented leisure is the smallest. Hypothesis H2 holds true. Among the three leisure activity categories, the model exhibits the strongest explanatory power for the entropy value of learning-oriented leisure activities and the weakest for social-oriented leisure activities.

**Figure 3:**
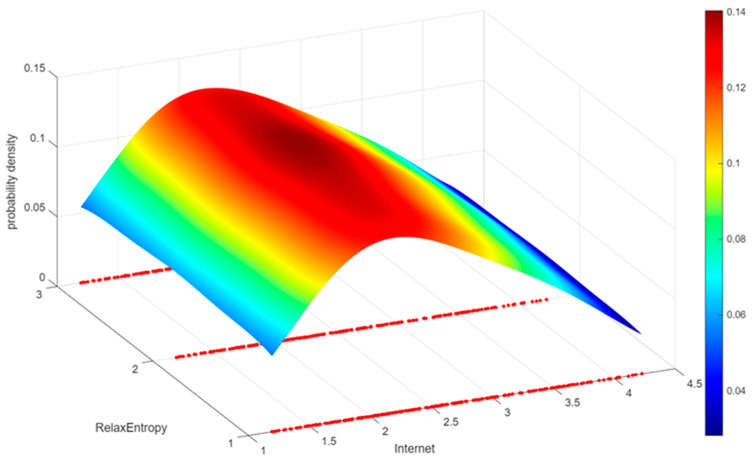
Relationship between Internet penetration, leisure activity growth orientations, and leisure entropy (The red dots denote the Internet penetration distribution across different types of leisure entropy).

Figure 3 presents the kernel density plots for internet penetration, leisure activity growth orientation, and leisure entropy. As internet penetration increases, leisure entropy first rises and then declines. The entropy value trends for leisure activities with different growth orientations are largely consistent, indicating that growth orientation does not significantly moderate entropy values.

## 5. Discussion

By introducing entropy theory into the realm of leisure activities, we constructed the concept of leisure activity entropy, derived the logic governing its changes, and proposed that leisure entropy enters a steady state when the entropy-increasing effect of external resource inputs balances the entropy-reducing effect of internal path dependencies. Through a macro–micro integrated framework and multi-method empirical testing, we derive five core conclusions on how internet penetration influences leisure activity entropy. Its underlying mechanisms and intrinsic logic can be further explained across three dimensions:

At the micro level, internet penetration increases leisure activity entropy. Empirical results show that a 1% increase in the frequency of internet use raises individual leisure entropy by 0.37%, with education and income amplifying this effect. This finding does not contradict existing research concluding that “the internet leads to fragmented leisure.” Rather, it stems from differing measurement perspectives. Previous studies focused on time allocated to individual leisure activities, while our research employs entropy theory to measure the overall diversity of leisure activities. Internet penetration satisfies individuals’ motivation for diversity by reducing costs and expanding accessibility, thus exhibiting an overall increase in entropy.

At the macro-level, internet penetration may exhibit an inverted U-shaped effect on internet influence. Macro internet penetration’s impact on regional leisure entropy may follow an inverted U-curve (with an inflection point at embedded software revenue of 3.03). The internet industry’s development may undergo phased transitions between an “innovation dividend period” and a “monopoly lock-in period.” During the early stages of industrial development, the universalization of the internet drives the expansion of digital leisure service supply, increasing regional leisure entropy. Later, as a few platforms establish monopolies, algorithmic homogenization may compress leisure choice space, causing entropy values to decline.

The impact of internet penetration on leisure activity entropy follows a one-way transmission system: “macro-level supply → micro-level constraints → decision feedback → macro-level steady state.” The mediation effect reveals that macro-level internet penetration generates an indirect effect of −0.033 through micro-level internet usage, while the direct effect is 0.140, resulting in a positive total effect. This indicates that technological spillovers from macro-level internet penetration directly enhance leisure diversity. However, increased usage frequency due to reduced micro-level usage costs paradoxically produces a weak negative mediating effect as some groups become locked into single leisure pathways. This contradictory outcome fills a gap in understanding macro–micro transmission mechanisms.

This paper makes three marginal academic contributions: 1. It constructs a theoretical framework for analyzing leisure activities under entropy theory. It clarifies the measurement formula and steady-state conditions for leisure entropy, resolving the challenge of accurately quantifying leisure diversity. This enriches the application of entropy theory in socioeconomic systems (particularly utility functions) and provides theoretical tools for the quantitative shift in leisure research. 2. It establishes a macro–micro integrated theoretical model of how internet penetration affects leisure activity entropy. Addressing the limitations of existing research that separates macro and micro perspectives, it integrates micro-level individual behavioral decision models with macroeconomic growth models. This creates a transmission system of “macro supply → micro constraints → decision feedback → macro steady state,” resolving the theoretical contradiction between “macro entropy increase” and “micro entropy decrease.” It forms a complete theoretical chain demonstrating how digital technology influences leisure well-being. 3. Revealed the nonlinear effects and effective configuration conditions of internet penetration on leisure entropy. A nonlinear model confirmed the inverted U-shaped effect of macro-level internet penetration. Using fs-QCA, seven effective configurations for increasing leisure entropy were identified, clarifying the core roles of education, income, and internet penetration. This expands the theoretical boundaries of the relationship between digital technology and leisure behavior, providing a configuration theory perspective for enhancing leisure well-being under complex conditions.

This study has several limitations that provide clear directions for future research. 1. Sample and geographical limitations exist. Our theoretical model was validated using Chinese sample data. However, the hypothesis linking individual leisure activity diversity to utility maximization may not hold in other cultural contexts. Additionally, China’s unique digital infrastructure and leisure culture may limit the direct generalizability of our findings to other countries. Future cross-national comparative studies could validate the cross-cultural applicability of the entropy theory framework and macro-micro models, revealing differentiated relationships between internet penetration and leisure entropy under varying institutional and cultural settings. 2. Robustness limitations in leisure entropy measurement. Existing measures convert self-reported activity frequencies into daily occurrence probabilities using weights arbitrarily set based on median time proportions. They also assume “individuals engage in only one leisure activity at a time,” potentially introducing bias in scenarios where multitasking leisure occurs. Given Shannon entropy’s sensitivity to input probability distributions, the subjectivity of these weighting assumptions may compromise the validity of leisure entropy measurements, thereby undermining the reliability of core findings. Future studies should replace self-reported frequency data with objective time-log data to directly capture actual duration shares of various leisure activities, ensuring the robustness of both leisure entropy measurements and regression results. 3. The validation of the inverted U-shaped effect has limitations. The macro-level inverted U-shaped impact of internet penetration on regional leisure entropy is currently supported solely by the regression results of the quadratic term of internet penetration, without incorporating direct measurement indicators such as platform market concentration. Consequently, the relevant inferences carry a degree of speculative nature. Future research could introduce direct indicators, such as the platform monopoly index, to strengthen the causal identification of the inverted U-shaped relationship.

## 6. Conclusions

Leisure activities in the digital age exhibit contradictory characteristics of “diversification expansion” and “monotonic lock-in.” Existing research struggles to accurately interpret the impact of internet penetration on leisure diversity due to fragmented macro-micro perspectives and insufficient theoretical tools. This study employs entropy theory to quantitatively measure the diversification of leisure activities, constructing a leisure activity entropy index. Building upon this foundation, a three-tier theoretical framework—“micro-individual—macro-regional—macro–micro linkage”—is established, quantifying leisure activity entropy as the equilibrium of leisure time allocation at the individual/regional level. This study systematically examines the impact and transmission mechanisms of internet penetration on leisure activity entropy using microdata from the China General Social Survey (CGSS) 2010–2023 and macro panel data from the China Economic and Financial Research Database. Employing benchmark regression, mediation effects, and nonlinear models, the research confirms the following: 1. At the micro level, internet usage significantly increases individual leisure entropy, with education and income amplifying this effect. 2. At the macro-level, internet industry development may exert an inverted U-shaped influence on regional leisure entropy. 3. Macro-level internet penetration transmits leisure entropy through micro-level internet usage. 4. Internet penetration exerts the strongest entropy-increasing effect on learning-oriented leisure activities, followed by relaxation-oriented leisure, while its impact on social-oriented leisure is the weakest. This paper makes two key academic contributions: 1. It constructs a theoretical framework for analyzing leisure activities under entropy theory. 2. It establishes a macro–micro integrated theoretical model of how internet penetration influences the entropy of leisure activities. This study represents a successful attempt to introduce physics’ entropy theory into social science leisure research, providing a model for interdisciplinary approaches to addressing leisure well-being issues. Future research can build upon this theoretical framework, further integrating perspectives from sociology, psychology, and other disciplines to explore the evolutionary patterns of leisure entropy, ultimately constructing a systematic theoretical framework for enhancing leisure utility in the digital age.

## Figures and Tables

**Table 1:** Variable characteristics table.

Variable Name	Micro Data		Macro Data	
	Mean	Standard Deviation	Mean	Standard Deviation
$I_{i}^{\rho -1}$	2.560	1.672		
Ii			198.499	494.797
$S_{i}^{\rho -1}$	0.570	0.210		
ESr			0.569	0.076
Year	1965.160	16.002	1965.596	4.398
Education	4.020	1.617	3.966	0.581
Income	32,839.600	155,609.695	30,624.127	22,021.524
Politics	1.410	0.988	1.403	0.167
Health	3.580	1.067	3.550	0.263
Happiness	3.860	0.827	3.876	0.176
Social status	4.240	1.701	4.256	0.374
Religious	1.502	1.323	1.886	1.184
pre-Income			20,024.461	9674.546
GDP			25,186.772	19,610.016
People			4705.207	2685.057
Sample size	62,837		234	

**Table 2:** Results of the benchmark regression table at the micro level.

Variable	Regression Results Without Controlled Variables			Regression Results with Controlled a Portion of the Control Variables			Regression Results with Controlled Variables		
	Coeff.	t	*p*.	Coeff.	t	*p*.	Coeff.	t	*p*.
constant	0.000	0.003	1.000	0.000	0.000	1.000	0.000	0.000	1.000
$I_{i}^{\rho -1}$	0.552	0.003	0.000	0.371	87.741	0.000	0.372	87.826	0.000
Year				0.012	2.821	0.005	0.015	3.595	0.000
Education				0.246	60.570	0.000	0.248	61.063	0.000
Income				0.008	2.481	0.013	0.008	2.373	0.018
Politics				0.054	15.833	0.000	0.053	15.710	0.000
Social status				0.092	28.209	0.000	0.083	24.697	0.000
Health				0.07	20.187	0.000	0.063	18.119	0.000
Religious							0.035	11.236	0.000
Happiness							0.033	9.850	0.000
R^2^	0.305			0.382			0.384		

**Table 3:** Benchmark regression table at the macro-level.

Variable	Regression Results Without Controlled Variables			Regression Results with Controlled a Portion of the Control Variables			Regression Results with Controlled Variables		
	Voeff.	t	*p*	coeff.	t	*p*.	Voeff.	t	*p*
Constant	0.000	0.000	1.000	0.000	0.000	1.000	0.000	0.000	1.000
$I_{i}$	0.247	3.877	0.000	0.114	2.114	0.036	0.107	2.032	0.043
Year				0.231	5.606	0.000	0.243	6.053	0.000
Education				0.637	11.205	0.000	0.556	9.415	0.000
Income				0.066	1.201	0.231	0.066	1.251	0.212
Politics				0.107	2.131	0.034	0.184	3.523	0.001
Social status				−0.096	−1.991	0.048	0.232	5.775	0.000
Health				−0.120	−2.906	0.004	−0.077	−1.844	0.067
pre-Income				−0.096	−1.991	0.048	−0.102	−2.129	0.034
GDP				0.016	0.169	0.866	0.036	0.394	0.694
People				−0.025	−0.364	0.716	−0.072	−1.079	0.282
Happiness							−0.137	−3.634	0.000
Religious							−0.091	−2.304	0.022
R^2^	0.061			0.751			0.77		

**Table 4:** Results of robustness test at the micro level.

Variable	Regression Results Without Controlled Variables			Regression Results with Controlled Variables		
	Coeff.	t	*p*	Coeff.	t	*p*
Constant	0.000	0.000	1.000	0.000	0.000	1.000
$I_{i}^{\rho -1}$	0.289	75.729	0.000	0.115	22.903	0.000
Year				0.085	17.165	0.000
Education				0.164	34.153	0.000
Religious				−0.006	−1.640	0.101
Income				0.008	2.018	0.044
Politics				0.054	13.291	0.000
Happiness				−0.018	−4.416	0.000
Social status				0.059	14.893	0.000
Health				0.072	17.325	0.000
R^2^	0.084			0.133		

**Table 5:** Endogenous test results.

Variable	Robustness Test 1			Robustness Test 2		
	Coeff.	t	*p*.	Coeff.	t	*p*.
constant	0	0.624	0.534	0	1.014	0.313
I_i1_				0.13	1.676	0.097
I_i2_	0.18	2.131	0.034			
Year	0.152	2.406	0.017	0.253	4.678	0
Education	0.342	3.618	0	0.45	5.058	0
Religious	−0.232	−4.211	0	−0.017	−0.324	0.747
Income	0.171	2.318	0.021	0.136	2.234	0.028
Politics	0	0.001	0.999	0.257	3.597	0
Happiness	−0.425	−8.508	0	−0.157	−3.561	0.001
Social status	0.105	1.886	0.061	0.212	4.029	0
Health	0.195	3.369	0.001	−0.01	−0.178	0.859
pre-Income	−0.076	−1.257	0.21	−0.125	−2.148	0.034
GDP	0.069	0.563	0.574	0.066	0.48	0.632
People	0.239	2.608	0.01	−0.136	−1.411	0.161
R^2^	0.628			0.843		

**Table 6:** Influence of Internet penetration on leisure activity entropy.

Variable	$I_{i}$—>$I_{i}^{\rho -1}$				$I_{i}$—>$ES_{r}$				$I_{i}\&I_{i}^{\rho -1}$—>$ES_{r}$			
	Coeff.	*p*	LLCI	ULCI	Coeff.	*p*	LLCI	ULCI	Coeff.	*p*	LLCI	ULCI
Constant	0.000	1.000	−0.048	0.048	0.000	1.000	−0.063	0.063	0.000	1.000	−0.063	0.063
Ii	−0.156	0.000	−0.234	−0.078	0.107	0.043	0.003	0.211	0.140	0.010	0.033	0.246
$I_{i}^{\rho -1}$									0.210	0.019	0.035	0.384
Year	0.390	0.000	0.330	0.449	0.244	0.000	0.164	0.323	0.162	0.002	0.058	0.266
Education	0.397	0.000	0.310	0.485	0.556	0.000	0.440	0.673	0.473	0.000	0.338	0.608
Income	0.107	0.007	0.029	0.185	0.066	0.212	−0.038	0.170	0.044	0.413	−0.061	0.148
Politics	−0.005	0.892	−0.082	0.072	0.184	0.001	0.081	0.286	0.185	0.000	0.083	0.287
Social status	0.050	0.098	−0.009	0.110	0.232	0.000	0.153	0.311	0.221	0.000	0.143	0.300
Happiness	−0.012	0.675	−0.068	0.044	−0.137	0.000	−0.211	−0.063	−0.135	0.000	−0.208	−0.061
Health	−0.053	0.092	−0.114	0.009	−0.077	0.067	−0.158	0.005	−0.066	0.115	−0.147	0.016
pre-Income	0.191	0.000	0.120	0.262	−0.102	0.034	−0.196	−0.008	−0.142	0.005	−0.241	−0.043
GDP	0.377	0.000	0.240	0.513	0.036	0.694	−0.146	0.219	−0.042	0.663	−0.234	0.150
People	−0.165	0.001	−0.263	−0.066	−0.072	0.282	−0.203	0.059	−0.037	0.582	−0.170	0.096
Religious	−0.110	0.000	−0.168	−0.052	−0.091	0.022	−0.168	−0.013	−0.067	0.094	−0.147	0.012
R^2^	0.871				0.771				0.776			

**Table 7:** Mediating effect coefficient table.

Effect Type	Effect	se	t	*p*	LLCI	ULCI
Direct effect	0.140	0.054	2.587	0.010	0.033	0.246
Indirect effect	−0.033	0.018			−0.076	−0.005
Total effect	0.107	0.053	2.032	0.043	0.003	0.211

**Table 8:** Inverted U-shaped inspection result table.

Variable	Regression Results Without Controlled Variables			Regression Results with Controlled Variables		
	Coeff.	t	*p*	Coeff.	t	*p*
Constant	0.077	1.037	0.301	0.066	1.702	0.090
Ii	0.545	3.423	0.001	0.400	3.560	0.000
$I_{i}^{2}$	−0.078	−1.924	0.056	−0.066	−2.938	0.004
Year				0.249	6.283	0.000
Education				0.538	9.222	0.000
Religious				−0.099	−2.564	0.011
Income				0.088	1.677	0.095
Political				0.189	3.682	0.000
Happiness				−0.121	−3.239	0.001
Social Hierarchy				0.222	5.598	0.000
Health				−0.080	−1.947	0.053
pre-Income				−0.085	−1.787	0.075
GDP				−0.058	−0.602	0.548
People				−0.046	−0.701	0.484
R^2^	0.084			0.779		

**Table 9:** Heterogeneity test results.

Variable	Social-Oriented Leisure			Relaxation-Oriented Leisure			Learning-Oriented Leisure		
	Coeff.	t	*p*	Coeff.	t	*p*	Coeff.	t	*p*
Constant	0.000	0.000	1.000	0.000	0.000	1.000	0.000	0.000	1.000
$I_{i}^{\rho -1}$	0.056	10.465	0.000	0.123	23.368	0.000	0.221	50.227	0.000
Year	0.016	3.033	0.002	−0.204	−39.226	0.000	−0.025	−5.834	0.000
Religious	0.007	1.889	0.059	0.026	6.607	0.000	0.027	8.185	0.000
Education	−0.013	−2.545	0.011	0.066	13.091	0.000	0.353	83.507	0.000
Income	−0.002	−0.460	0.646	−0.009	−2.260	0.024	0.010	2.907	0.004
Politics	0.020	4.642	0.000	0.013	3.152	0.002	0.132	37.265	0.000
Happiness	0.075	17.010	0.000	−0.031	−7.167	0.000	0.029	8.001	0.000
Health	0.042	9.862	0.000	0.093	22.426	0.000	0.034	9.776	0.000
Social status	0.067	15.995	0.000	0.019	4.498	0.000	0.062	17.851	0.000
R^2^	0.025			0.046			0.333		

## Data Availability

The original data presented in the study are openly available in Mendeley Data at DOI:10.17632/c75kyhzbp9.2, accessed on 17 December 2025.

## Appendix A. Theoretical Derivation Process

### Appendix A.1. Micro Individual Behavior Decision Model

At the micro level, this paper focuses on the decision-making logic of individual internet usage and leisure entropy. Based on the “diversity-seeking theory” and neoclassical consumer choice theory, it constructs a utility function incorporating leisure entropy preferences. By integrating time and budget constraints, it derives optimal decisions and subsequently proposes a core proposition.

#### Appendix A.1.1. Improved Utility Function

The preference for diversity in leisure activities traces back to behavioral economics’ Diversity Seeking Theory, which posits that individuals possess an endogenous need for variety in consumption and behavioral decisions. Neoclassical consumer choice theory also assumes consumers prefer “diversified” combinations when constructing utility functions. Iso-Ahola noted that individuals with access to a wider range of leisure activities possess greater choice during their free time and are more likely to find alternatives when current leisure pursuits cease to provide positive experiences and rewards [45]. Empirical findings by Lee corroborate this observation [46]. Furthermore, leisure activities and internet usage can enhance subjective well-being [47,48].

Based on these findings, we incorporate leisure diversity and internet usage into microeconomic utility functions. This paper posits that individual i, constrained by time and budget, seeks to maximize utility incorporating leisure entropy preferences. We employ a CES-Cobb–Douglas hybrid utility function to examine the relationship between internet usage and leisure diversity [49]:

(A1) $$U_{i}={\left[\omega_{i}S_{i}^{\rho }+\left(1-\omega_{i}\right)I_{i}^{\rho }\right]}^{1/\rho }\cdot C_{i}^{\gamma },\;\rho <0,\;\omega_{i}\in \left(0,1\right),\;\gamma >0$$

The theoretical implications and economic meanings of each variable are as follows. $S_{i}$ represents the leisure activity entropy value for individual i, measuring the diversity of leisure time allocation. Its utility contribution stems from the individual’s motivation to seek diversity. $I_{i}$ represents the intensity of internet usage for individual i, serving as a proxy variable for internet penetration at the micro level. $C_{i}$ denotes consumption of non-leisure goods, satisfying basic living needs. $\omega_{i}$ is the preference weight for leisure diversity; higher-educated groups exhibit stronger cognition and demand for behavioral diversity [50]; hence, leisure diversity is positively influenced by individual education level ($\frac{\partial \omega_{i}}{\partial Edu_{i}}>0$). ρ (where ρ < 0) represents the substitution elasticity parameter, indicating a complementary relationship between $S_{i}$ and $I_{i}$ (decreasing marginal rate of substitution), consistent with the integrated nature of “Internet + leisure” (e.g., commenting in online communities simultaneously relies on internet access and leisure time) [51]. γ denotes the utility elasticity of consumption, reflecting an individual’s emphasis on basic consumption.

#### Appendix A.1.2. Constraint Conditions

Individuals face two constraints: time constraints and budget constraints.

Each person has a fixed total of 24 h per day, which must be allocated among work, sleep, pure leisure, and internet usage. Therefore, this study incorporates a time constraint into the model, defined as:

(A2) $$T_{i}^{\mathrm{work}}+T_{i}^{\mathrm{leis}}+I_{i}+S=T_{0}=24$$

In the equation, $T_{i}^{\mathrm{work}}$ represents labor supply time, $T_{i}^{\mathrm{leis}}$ denotes pure leisure time, $I_{i}$ signifies internet usage time, and S indicates sleep time.

Individual labor income must cover commodity consumption, internet usage costs, and participation costs for various leisure activities. Therefore, this study incorporates a budget constraint into the model, defined as:

(A3) $$W_{i}T_{i}^{\mathrm{work}}=P_{C}C_{i}+P_{I}I_{i}+\sum_{k=1}^{K}P_{k}T_{ik}$$

(A4) $$T_{i}^{\mathrm{leis}}=\sum_{k=1}^{K}T_{ik}$$

In the equation, $W_{i}$ represents the hourly wage, $P_{I}$ denotes the unit cost of internet usage, and $P_{k}$ signifies the unit time cost of the kth type of leisure activity.

#### Appendix A.1.3. Optimal Decision Making and Lagrange Multiplier Derivation

To maximize utility incorporating leisure entropy preferences, this study constructs the following Lagrangian function:

(A5) $$L_{i}=\omega_{i}S_{i}^{\rho }+\left(1-\omega_{i}\right)I_{i}^{\rho }+\gamma lnC_{i}-\lambda_{i}\left(P_{C}C_{i}+P_{I}I_{i}+\sum_{k}P_{k}T_{ik}-W_{i}T_{i}^{\mathrm{work}}\right)-\mu_{i}\left(T_{i}^{\mathrm{work}}+T_{i}^{\mathrm{leis}}+I_{i}-24\right)$$

This study calculates the partial derivatives of the decision variables $C_{i}$, $I_{i}$, $T_{ik}$, and $T_{i}^{work}$ and sets them equal to zero, yielding the following core first-order conditions:

By taking a partial derivative of $C_{i}$:

(A6) $$\frac{\partial L_{i}}{\partial C_{i}}=\frac{\gamma }{C_{i}}-\lambda_{i}P_{C}=0$$

The optimal condition for obtaining commodity consumption is:

(A7) $$\frac{\gamma }{C_{i}}=\lambda_{i}P_{C}$$

(A8) $$C_{i}^{*}=\frac{\gamma }{\lambda_{i}P_{C}}$$

This indicates that the marginal utility of commodity consumption is equal to the marginal utility of unit income.

By taking a partial derivative of $I_{i}$:

(A9) $$\frac{\partial L_{i}}{\partial I_{i}}=\frac{\left(1-\omega_{i}\right)I_{i}^{\rho -1}}{\omega_{i}S_{i}^{\rho }+\left(1-\omega_{i}\right)I_{i}^{\rho }}-\lambda_{i}P_{I}-\mu_{i}=0$$

The best condition for sorting out the Internet usage is:

(A10) $$\frac{\left(1-\omega_{i}\right)I_{i}^{\rho -1}}{\omega_{i}S_{i}^{\rho }+\left(1-\omega_{i}\right)I_{i}^{\rho }}=\lambda_{i}P_{I}+\mu_{i}$$

By taking a partial derivative of $T_{ik}$:

(A11) $$\frac{\partial L_{i}}{\partial T_{ik}}=\frac{\omega_{i}S_{i}^{\rho -1}\cdot \frac{\partial S_{i}}{\partial T_{ik}}}{\omega_{i}S_{i}^{\rho }+\left(1-\omega_{i}\right)I_{i}^{\rho }}-\lambda_{i}P_{k}-\mu_{i}=0$$

The optimal condition for organizing leisure time is

(A12) $$\frac{\omega_{i}S_{i}^{\rho -1}\cdot \frac{\partial S_{i}}{\partial T_{ik}}}{\omega_{i}S_{i}^{\rho }+\left(1-\omega_{i}\right)I_{i}^{\rho }}=\lambda_{i}P_{k}+\mu_{i}$$

In the expression, $\frac{\partial S_{i}}{\partial T_{ik}}=\frac{1}{T_{i}^{leis}}\left(ln\frac{T_{i}^{leis}}{T_{ik}}-1\right)$ represents the marginal rate of change in leisure entropy with respect to the kth category of leisure time.

By taking a partial derivative of $T_{i}^{work}$:

(A13) $$\frac{\partial L_{i}}{\partial T_{i}^{work}}=\lambda_{i}W_{i}-\mu_{i}=0$$

The optimal condition for organizing labor supply is:

(A14) $$\lambda_{i}W_{i}=\mu_{i}$$

This indicates that the marginal income utility of labor time is equal to the marginal utility of time.

Substituting $\mu_{i}=\lambda_{i}W_{i}$ into the optimal conditions for internet usage and leisure time yields the utility maximization condition incorporating leisure entropy preferences under dual constraints of time and budget, as follows:

(A15) $$\frac{\left(1-\omega_{i}\right)I_{i}^{\rho -1}}{\omega_{i}S_{i}^{\rho }+\left(1-\omega_{i}\right)I_{i}^{\rho }}=\lambda_{i}\left(P_{I}+W_{i}\right)$$

(A16) $$\frac{\omega_{i}S_{i}^{\rho -1}\cdot \frac{\partial S_{i}}{\partial T_{ik}}}{\omega_{i}S_{i}^{\rho }+\left(1-\omega_{i}\right)I_{i}^{\rho }}=\lambda_{i}\left(P_{k}+W_{i}\right)$$

#### Appendix A.1.4. Key Proposition and Mechanism Explanation

Based on the derivation of optimal decisions outlined above, three core propositions emerge at the micro level, from which research hypotheses are deduced:

**Proposition** **A1.** *Increased frequency of internet use significantly elevates individual leisure activity entropy (i.e., under conditions where ρ < 0 and* $P_{I}$ *is given, *$\frac{\partial S_{i}^{*}}{\partial I_{i}}>0$*). The internet enhances leisure diversity through cost-reduction and accessibility expansion effects: on one hand, it lowers participation barriers for niche leisure activities; on the other, integrated leisure platforms broaden the range of leisure options.*

Based on Proposition A1, we propose Hypothesis H1: At the micro level, internet penetration significantly and positively influences the entropy value of individual leisure activities, meaning that higher internet penetration correlates with greater diversity in leisure activities.

**Proposition** **A2.**
*Increases in educational attainment and income amplify the internet’s entropy-increasing effect on leisure activities. Higher education strengthens individuals’ preference for diversity, while increased income eases budget constraints on high-cost leisure activities. Both factors reinforce the internet’s role in promoting leisure entropy.*

**Proposition** **A3.**
*The internet’s entropy-increasing effect is significantly greater for relaxation-oriented leisure (e.g., watching movies, listening to music) than for social-oriented leisure (e.g., offline gatherings), i.e.,* $\Delta S_{i}^{relax}>\Delta S_{i}^{social}$ *. Relaxation-oriented leisure activities exhibit greater online adaptability, with the internet substantially lowering participation costs and time barriers. Conversely, the core value of social leisure activities stems from offline interaction. The substitution effect of the internet may reduce the proportion of time spent on offline socializing, resulting in limited diversity enhancement.*

Based on Proposition A3, we propose Hypothesis H2: At the micro level, internet penetration significantly increases the entropy of relaxation-oriented leisure activities more than social leisure activities.

### Appendix A.2. Macroeconomic Growth Model

At the macro-level, this paper examines the causal relationship between regional internet industry development and leisure entropy. By modifying the traditional neoclassical growth model to incorporate leisure entropy into the social welfare function, it constructs a composite welfare-output model. Drawing on endogenous growth theory, the paper derives core propositions and hypotheses.

#### Appendix A.2.1. Composite Well-Being Output Function

Traditional neoclassical growth theory focuses solely on economic output [52]. However, Amartya Sen’s capability theory and subjective well-being theory emphasize that subjective well-being should encompass non-economic welfare dimensions such as leisure [53]. In the digital economy era, the development level of regional leisure activities has become a critical factor influencing the quality of social development [36]. Therefore, this paper incorporates regional leisure entropy $S_{r}$ into the social welfare function to construct a composite well-being output function.

(A17) $$Y_{r}^{\mathrm{total}}=\theta Y_{r}+\left(1-\theta \right)S_{r},\;\theta \in \left(0,1\right)$$

In the equation, $Y_{r}$ represents the economic output of region r, $S_{r}$ denotes the regional leisure entropy (the average of micro-level individual leisure entropy), and θ indicates the societal weighting preference between economic output and behavioral diversity. The closer θ approaches 1, the greater the societal emphasis on economic output; the closer θ approaches 0, the greater the societal emphasis on behavioral leisure well-being.

#### Appendix A.2.2. Production Functions and Technological Progress

Regional economic output $Y_{r}$ employs an improved Cobb–Douglas production function [54], with embedded software revenue ($ES_{r}$) endogenized as the core driver of technological progress. This aligns with the endogenous growth theory hypothesis that “technological progress is the core engine of economic growth” [55], expressed as:

(A18) $$Y_{r}=A_{r}K_{r}^{\alpha }L_{r}^{1-\alpha },\;A_{r}=A_{0}\cdot ES_{r}^{\eta },\;\eta >0$$

In the equation, $K_{r}$ represents regional leisure-related capital stock (e.g., cultural venues, digital infrastructure investment), reflecting the level of capital investment in the leisure industry. $L_{r}$ denotes regional leisure-related labor input (e.g., cultural tourism practitioners, digital service personnel). $A_{r}$ signifies regional total factor productivity, measuring the level of technological progress. $A_{0}$ represents the initial technological level, indicating the foundational endowment of regional technological development. $ES_{r}$ denotes embedded software revenue, serving as a proxy variable for internet penetration at the macro-level due to embedded software’s critical role in enabling internet penetration. η > 0: the coefficient representing embedded software revenue’s contribution to technological progress, reflecting the spillover effects of digital technology. α (α ∈ [0, 1]) is the capital-output elasticity, consistent with the parameter constraints of the neoclassical production function.

#### Appendix A.2.3. Capital Accumulation and Steady-State Analysis

According to the capital accumulation rules of neoclassical growth theory, the accumulation equation for regional leisure-related capital is:

(A19) $${\dot{K}}_{r}=sY_{r}-\delta K_{r}$$

Where s denotes the regional savings rate (the proportion of output converted into leisure industry investment), δ represents the capital depreciation rate, and ${\dot{K}}_{r}$ signifies the rate of change in the capital stock.

At steady state (${\dot{k}}_{r}=0$, $k_{r}=K_{r}/L_{r}$ being the regional per capita leisure capital), the steady-state per capita capital stock can be derived as:

(A20) $$k_{r}^{*}={\left(\frac{sA_{0}}{\delta +n}\right)}^{\frac{1}{1-\alpha -\eta }}ES_{r}^{\frac{\eta }{1-\alpha -\eta }}$$

Here, n represents the regional population growth rate. The precondition for steady-state existence is 1 − α − η > 0 (i.e., α + η < 1), which ensures that capital accumulation does not expand indefinitely, aligning with the convergent characteristics of the economic system.

#### Appendix A.2.4. Macro Propositions and Mechanistic Explanations

Based on a composite welfare production function and steady-state analysis, two core propositions are derived at the macro-level, along with corresponding hypotheses:

**Proposition** **A4.**
*Internet penetration significantly increases regional leisure entropy. The mechanism involves two pathways: First, the technology spillover pathway—internet penetration generates technology spillover effects, boosting residents’ income and thereby relaxing budget constraints on leisure choices, thus enhancing the information entropy of leisure activities. Second, the supply expansion pathway—internet penetration promotes innovation in digital leisure service supply, directly broadening regional leisure choice sets and driving up the information entropy of leisure activities.*

Based on Proposition A4, we propose Hypothesis H3: At the macro-level, regional internet industry development significantly and positively influences regional leisure entropy. That is, higher levels of internet industry development correlate with greater overall diversity (entropy) in regional leisure activities.

**Proposition** **A5.**
*Increased per capita disposable income provides residents with greater resources to participate in diverse leisure activities. Population growth reduces the marginal cost of digital services, enabling more people to access and engage in leisure activities at lower costs. Regional GDP growth provides financial support for digital infrastructure development, thereby enhancing the role of technological progress in promoting behavioral diversity.*

### Appendix A.3. Macro–Micro Linkage Model

Micro models and macro models, respectively, explain the independent logics of individual decision-making and regional development, yet they share an inherent transmission relationship. This paper constructs a unidirectional transmission system—“macro supply → micro constraints → decision feedback → macro steady state”—based on neoclassical supply-demand transmission theory and industrial organization theory. It derives the core proposition of macro–micro linkage and proposes corresponding hypotheses.

**Proposition** **A6.**
*Macro-level internet penetration influences leisure activity entropy by affecting micro-level internet penetration. Macro-level internet penetration indirectly impacts leisure activity entropy through micro-level penetration, with transmission pathways comprising direct and indirect effects. Regarding direct effects, growth in embedded software revenue promotes the universalization of digital leisure technologies in a region. Even if individuals do not directly increase internet usage, they can enjoy diversified leisure services through technological spillovers, directly enhancing regional leisure entropy. Regarding indirect effects, growth in embedded software revenue intensifies competition among regional digital service platforms, forcing down internet usage costs (* $P_{I}$ *). The loosening of micro-level individual constraints on internet usage costs encourages increased internet usage frequency. This, in turn, elevates the entropy value of leisure activities through the micro-level main effect of Proposition A1, ultimately aggregating into growth in regional leisure entropy (* $S_{r}$ *).*

Based on Proposition A6, we propose Hypothesis H5: Macro-level internet industry development indirectly influences leisure activity entropy through the mediating effect of micro-level internet penetration, meaning micro-level internet penetration serves as the mediating variable for macro-level internet penetration’s impact on leisure activity entropy.

**Proposition** **A7.**
*Macro-level internet penetration exhibits a double-edged sword effect, influencing leisure entropy in an inverted U-shaped pattern. According to the monopoly suppression hypothesis in industrial organization theory, during the late stages of macro-level internet penetration, a few platforms may form market monopolies. These monopolies suppress the diversity of leisure services through algorithmic homogenization, service exclusivity, and other means [56]. A nonlinear model incorporating a quadratic term for embedded software revenue can capture this effect:*

(A21)
$$I_{i}=\beta_{1}ES_{r}+\beta_{2}ES_{r}^{2}+\epsilon_{i},\;\beta_{1}>0,\;\beta_{2}<0$$

The corresponding inverted U-shaped effect can be expressed as:

(A22) $$\frac{\partial S_{r}}{\partial ES_{r}}={\left(\frac{\partial S_{r}}{\partial ES_{r}}\right)}^{direct}+{\left(\frac{\partial S_{r}}{\partial ES_{r}}\right)}^{indirect}$$

Among these, the direct effect is positive during the initial phase of embedded software revenue but weakens later due to diminishing monopolistic power. The indirect effect exhibits a shift from positive to negative as embedded software revenue grows, ultimately resulting in an inverted U-shaped total effect of embedded software revenue on the entropy value of leisure activities.

Based on Proposition A7, we propose Hypothesis H4: At the macro-level, the impact of internet industry development on regional leisure entropy follows an inverted U-shaped pattern. Specifically, during the early stage of industry development (when embedded software revenue is below the threshold), it increases regional leisure entropy, while in the later stage (when embedded software revenue exceeds the threshold), it decreases regional leisure entropy.
